# 

**Published:** 1889-08

**Authors:** 


					﻿io cts. a copy
AUGUST, 188?.
$1.00 per y ear.
WALLS s
sJovrnalj
WEALT W
ESTABLISHED 1854
IE- 3o
8?
OFFICE! 206 BROADWAY. EVENING POST BUILDING, Boom 11. N. Y.
Entered as Second-class Matter at the Post-Office, New York.
				

